# Perceptual learning based on a temporal stimulus enhances visual function in adult amblyopic subjects

**DOI:** 10.1038/s41598-023-34421-3

**Published:** 2023-05-11

**Authors:** Auria Eisen-Enosh, Nairouz Farah, Uri Polat, Yossi Mandel

**Affiliations:** 1 School of Optometry and Vision Science, Bar-Ilan University, Ramat Gan, Israel; 2 Bar-Ilan Institute for Nanotechnology and Advanced Materials (BINA), Bar-Ilan University, Ramat Gan, Israel; 3 The Leslie and Susan Gonda (Goldschmied) Multidisciplinary Brain Research Center, Bar-Ilan University, Ramat Gan, Israel

**Keywords:** Learning and memory, Visual system

## Abstract

Studies have shown that Perceptual Learning (PL) can lead to enhancement of spatial visual functions in amblyopic subjects. Here we aimed to determine whether a simple flickering stimulus can be utilized in PL to enhance temporal function performance and whether enhancement will transfer to spatial functions in amblyopic subjects. Six adult amblyopic and six normally sighted subjects underwent an evaluation of their performance of baseline psychophysics spatial functions (Visual acuity (VA), contrast sensitivity (CS), temporal functions (critical fusion frequency (CFF) test), as well as a static and flickering stereopsis test, and an electrophysiological evaluation (VEP). The subjects then underwent 5 training sessions (on average, a total of 150 min over 2.5 weeks), which included a task similar to the CFF test using the method of constant stimuli. After completing the training sessions, subjects repeated the initial performance evaluation tasks. All amblyopic subjects showed improved temporal visual performance (CFF) in the amblyopic eye (on average, 17%, p << 0.01) following temporal PL. Generalization to spatial, spatio-temporal, and binocular tasks was also found: VA increased by 0.12 logMAR (p = 0.004), CS in backward masking significantly increased (by up to 19%, p = 0.003), and flickering stereopsis increased by 85 arcsec (p = 0.048). These results were further electrophysiologically manifested by an increase in VEP amplitude (by 43%, p = 0.03), increased Signal-to-Noise ratio (SNR) (by 39%, p = 0.024) to levels not different from normally sighted subjects, along with an improvement in inter-ocular delay (by 5.8 ms, p = 0.003). In contrast, no significant effect of training was found in the normally sighted group. These results highlight the potential of PL based on a temporal stimulus to improve the temporal and spatial visual performance in amblyopes. Future work is needed to optimize this method for clinical applications.

## Introduction

Amblyopia is decreased visual function in (usually) one eye resulting from abnormal development of the visual system in early stages of life^1^. Amblyopia can develop due to several factors, such as anisometropia or strabismus, both causing a poor retinal quality image, thus preventing normal visual cortex development during the "critical period"^1–4^. Clinically, amblyopia is usually manifested by a unilateral decrease in best-corrected visual acuity as well as other spatial-visual function abnormalities such as reduced contrast sensitivity, Vernier acuity^5–12^, and the presence of spatial crowding^13^. In addition to decreased spatial vision functions, amblyopic subjects also experience reduced temporal functions, such as temporal crowding, a longer reaction time, and reduced CFF thresholds^12,14–26^.

Amblyopia is associated with several temporal deficits^12,24,27^, which include impaired perception of motion-defined form^28,29^ and temporal integration^30^. Furthermore, there is an increased response latency to stimuli^31^ presented to the amblyopic eye, ^14^which was measured both psychophysically and electrophysiologically^15–18^.

Visual Perceptual Learning (PL) is a well-established tool for improving visual function for a wide range of tasks, especially in amblyopic subjects^32–34^. PL is obtained through practice and training, and relies on the remaining post critical period neural plasticity^35^, with the hypothesis that practicing challenging visual tasks around the threshold will lead to an improvement in the corresponding task.

The learning procedure is hypothesized^36^ to be mediated via synaptic mechanisms leading to modification of excitation inhibition balance^37–40^. It is widely agreed that there are three distinct mechanisms underlying PL: stimulus enhancement (amplification), external noise exclusion (filtering), and changes in gain control^41,42^.

The PL paradigms of the visual training often employ tasks from the spatial domain of the visual system (e.g., discrimination between different orientations, and discrimination between different contrasts)^33,41^; the result of these training sessions is mainly reflected in spatial function (visual acuity, contrast sensitivity, and Vernier discrimination). There are only a few studies that focused on training the temporal domain. Seitz et al*.* reported increased CFF thresholds following a motion-direction learning procedure (a rapid serial visual presentation (RSVP) letter-identification task)^43^. Another study showed that directional dot-motion and contrast sensitivity training also resulted in a significant improvement in CFF^44^. It is important to note that the task in the majority of those works was not purely temporal, but rather, it had definite spatial characteristics (e.g.^45,46^).

Successful perceptual learning is assessed by the transfer of acquired performance enhancements to modified forms of the same task or to different related tasks^34,39,41,47^. Numerous studies have shown that the improvement resulting from PL is not specific to the task of the training, but rather, it is transferred to the other eye and to other tasks such as contrast sensitivity, visual acuity^34,48^, reading speed^49^, and/or to visual and stereo acuity^33^.

Stereopsis is an important visual function^50–53^ that is highly dependent on the proper function of each eye separately and on the proper integration of the information at the spatiotemporal domains^54^. Stereo function significantly deteriorates under a flickering condition^27,55^ in amblyopes (as well as in normal subjects) because of inter-ocular temporal asynchrony; however, it can be significantly enhanced by inter-ocular re-synchronizing^27^. Therefore, we hypothesized that training in the temporal domain can significantly affect stereo function in general and flickering stereopsis in particular.

Moreover, it was demonstrated that the learning procedure in the spatial domain can transfer to the temporal domain performance, as is evident from an improvement in temporal parameters such as stimulus onset asynchrony (SOA), reaction time (RT), temporal modulation transfer functions (MTF)^56^, or CFF^44^ and temporal integration^49,57^. One possible explanation for the transfer is that training improves the processing speed, which is a common mechanistic pathway for improvement in other visual functions^57,58^.

Another important feature of amblyopia is an inter-ocular imbalance, which adversely affects binocular information integration, leading to reduced stereo vision^59^ and binocular summation (BS)^60–68^ (see recent review^59^). Therefore, recent training procedures are aimed at regaining inter-ocular balance, integration, and binocular functions^69–72^.

In the current study we hypothesized that a short training procedure given to the amblyopic eye with flickering stimuli in a temporal task of CFF will increase the visual temporal functions. We also investigated whether the increase in processing speed in the amblyopic eye will increase the inter-ocular synchronization and thus enhance binocular function performance and investigated whether it will be transferred to other visual functions.

To this end, we studied the effect of a short (5 sessions) PL procedure that utilized a customized dichoptic system to stimulate the eyes with a flickering stimulus in a CFF paradigm^26^. We evaluated CFF throughout the training sessions and also studied the effect of training on spatio-temporal functions using computerized contrast sensitivity tests, flickering stereopsis tests, and electrophysiological recordings of steady state visual evoked potentials (ssVEP).

Our results indicate that flickering stimulus-based PL elicited a significant improvement in both the temporal (CFF) and spatiotemporal (flickering stereopsis and backward masking (BM)) performance of amblyopic subjects. Moreover, the functional improvement was transferred to spatial function, which was evident in the improvement in VA. The effect of training was also manifested in the increased VEP amplitude and a significant increase in inter-ocular synchronization.

## Results

### Flickering perceptual learning elicited a significant increase in CFF in amblyopic subjects

Six amblyopic subjects underwent 5 training sessions, which included a task similar to the CFF test described in our previous study^73^ (see the “Methods” for more details).

**Figure 1:**
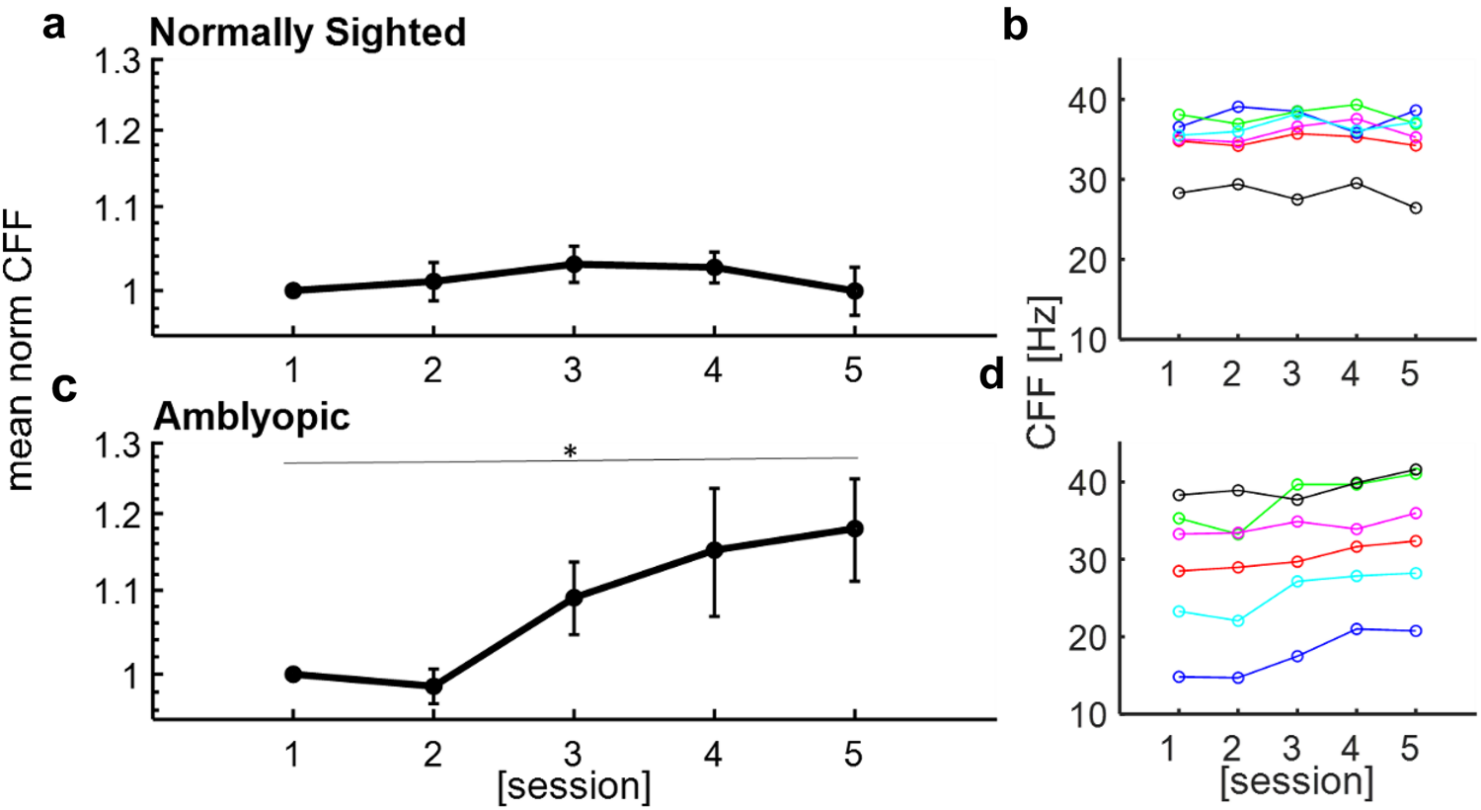
Effect of training on CFF thresholds. (**a**) Normalized (black) average CFF thresholds of the NDE in normally sighted subjects as a function of the training session. (**b**) Individual (color coded) CFF thresholds of NDE from all normally sighted subjects as a function of the training session. (**c**) Normalized (black) average CFF thresholds of the AE as a function of the training session. (**d**) Individual (color coded) CFF thresholds of AE as a function of the training session. Error bars refer to the standard error of the mean. Statistical significance was indicated: * p <  = 0.05.

Interestingly and in line with our hypothesis, the amblyopic subjects’ CFF threshold gradually increased throughout the training session (Fig. 1c, d); the average CFF during the last session was 18 percent higher than that of the first session CFF (22.8 ± 3.4 Hz vs 26.7 ± 3.15 Hz, paired two-tailed t-test *p* << 0.05, t(5) = − 8.12). Individual psychophysical curves of the first and fifth session of training amblyopic and normally sighted subjects are presented in Figs. S1 and S2, respectively, showing a training-induced right shift of the curve (indicating an increase in the CFF threshold). In contrast to the significant improvement in amblyopes, the thresholds of normal subjects did not improve during the training sessions (30.34 ± 0.5 vs 30.7 ± 0.7, paired two-tailed t-test *p* = 0.92, t(5) = − 0.1) (Fig. 1a, b).

**Figure 2:**
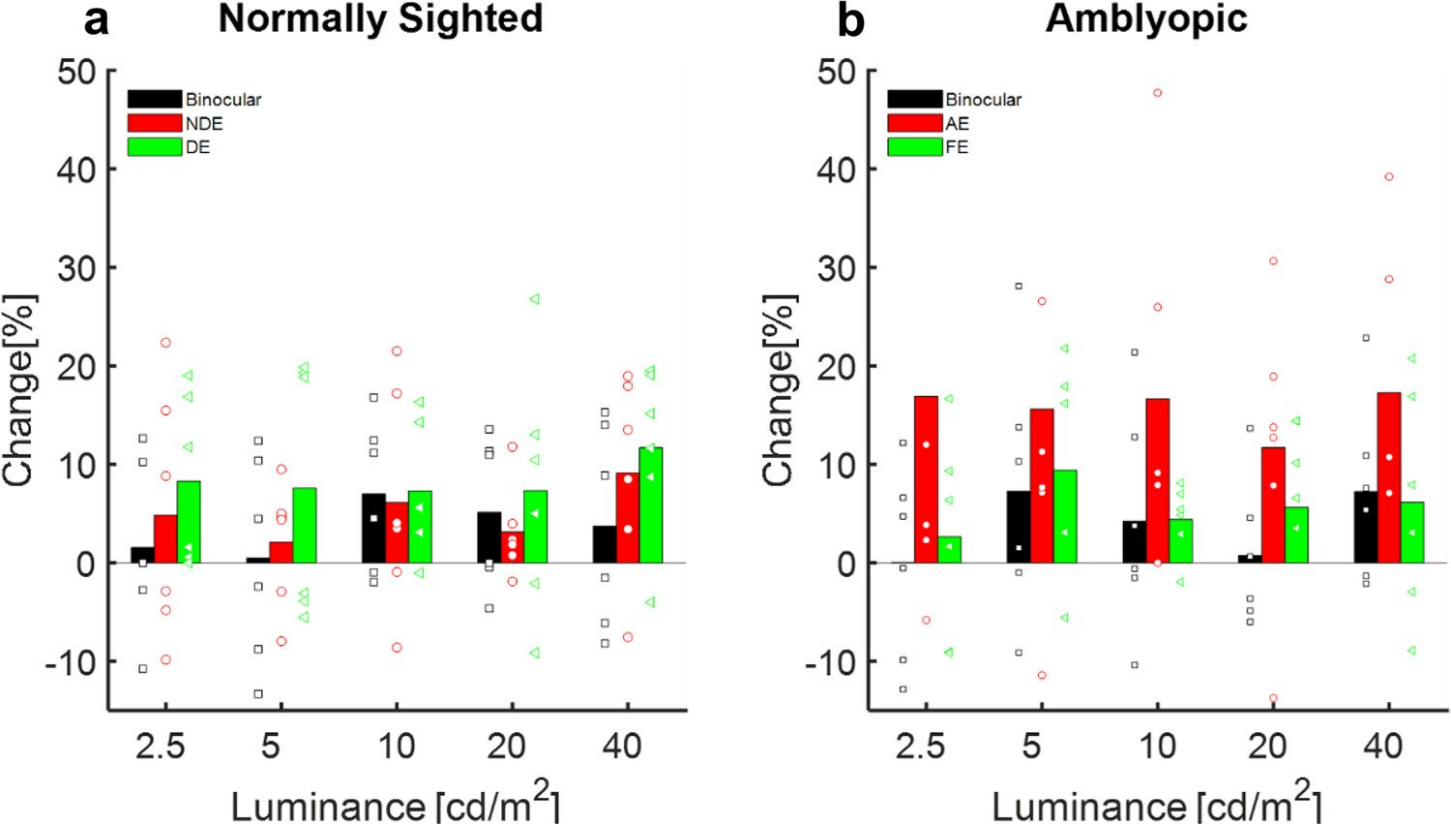
The effect of training on CF: percentage change in the CFF threshold after training, compared to baseline CFF. (**a**) Normally sighted subjects. Black bars denote the mean change in CFF under binocular viewing conditions; the red bars denote the mean change in the CFF threshold for the NDE, and green bars denote the mean change in the CFF threshold for the DE. Each black, square; red, circle and green, triangle denotes an individual’s change in CFF threshold under binocular viewing conditions, the NDE, and the DE, respectively. (**b**) Amblyopic subjects. Black bars denote the mean change in CFF under binocular viewing conditions, the red bars denote the mean change in CFF for the AE, and green bars denote the mean change in CFF for the FE. Each black square red circle, and green triangle denotes an individual’s change in CFF threshold under binocular viewing conditions, the AE, and the FE, respectively.

CFF was further evaluated at five different luminance levels before and after training, for both monocular and binocular viewing conditions by a dichoptic flicker stimulation system using the staircase paradigm^26,73^ (see the “Methods” for more details). These measurements further validated the significant increase in CFF thresholds following training (Fig. 2b) at all luminance levels (t-test: *p* << 0.01, repeated measure ANOVA *p* << 0.01, *F* (1,58) = 39.247). The largest improvement (17%) was observed at a luminance level of 10 cd/m^2^ (28.7 ± 3.4 vs 32.3 ± 3.18.

It is important to note that before training, the amblyopic eyes’ CFF threshold was significantly lower, compared with the fellow eye (FE) and compared with the non-dominant eye (NDE) of normally sighted subjects by a mean difference of 13.5% (27.3 ± 3.4 vs 30.1 ± 2.7, paired two-tailed t-test *p* << 0.01 t(4) = − 9) and 15%, (27.3 ± 3.4 vs 31.41 ± 2.5, two-tailed t-test *p* = 0.02 t(8) = − 2.4), respectively; repeated measure ANOVA *p* << 0.05, *F* = 18.84 and *p* << 0.01, *F* = 5.1, respectively), consistent with our results^26,27^and those of other studies^21,74,75^. In contrast, following training, the CFF threshold in the AE was not significantly different from the FE or the NDE of normal subjects (30.6 ± 5.1 vs 31.7 ± 3.3, paired two-tailed t-test *p* = 0.98 t(4) = − 2.8) and (30.6 ± 5.1 vs 33.02 ± 3.4 two-tailed t-test *p* = 0.13 t(8) = − 1.17, respectively); ANOVA f(9) = 1.46 p = 0.19, f(9) = 0.173 p = 0.1).

Although a small improvement trend was observed for both the FE of amblyopic subjects and normally sighted subjects, it was significantly lower compared with the improvement in amblyopic eyes (30.12 ± 2.7 vs 31.7 ± 3.3, paired two-tailed t-test *p* = 0.38 t(5) = − 0.9) and (31.4 ± 2.5 vs 33.02 ± 3.5 two-tailed t-test *p* = 0.37 t(8) = − 0.94 comparison before and after FE and NDE, respectively); thus, the difference between pre- and post-training was not statistically significant in normally sighted subjects (Fig. 2a) (individual changes can be seen in supplementary (Fig. S3).

### Training with a flickering stimulus significantly increased visual acuity in the amblyopic eye

As expected, the pre-training VA of AE in the amblyopic subjects was significantly lower than that of FE (0.38 ± 0.004 vs 0.02 ± 0.004, paired two-tailed t-test *p* << 0.01, t(5) = 7.75) and that of the NDE of normally sighted subjects (0.38 ± 0.004 vs − 0.06 ± 0.0008, paired two-tailed t-test *p* << 0.01,t(5) = 9.02).

**Figure 3:**
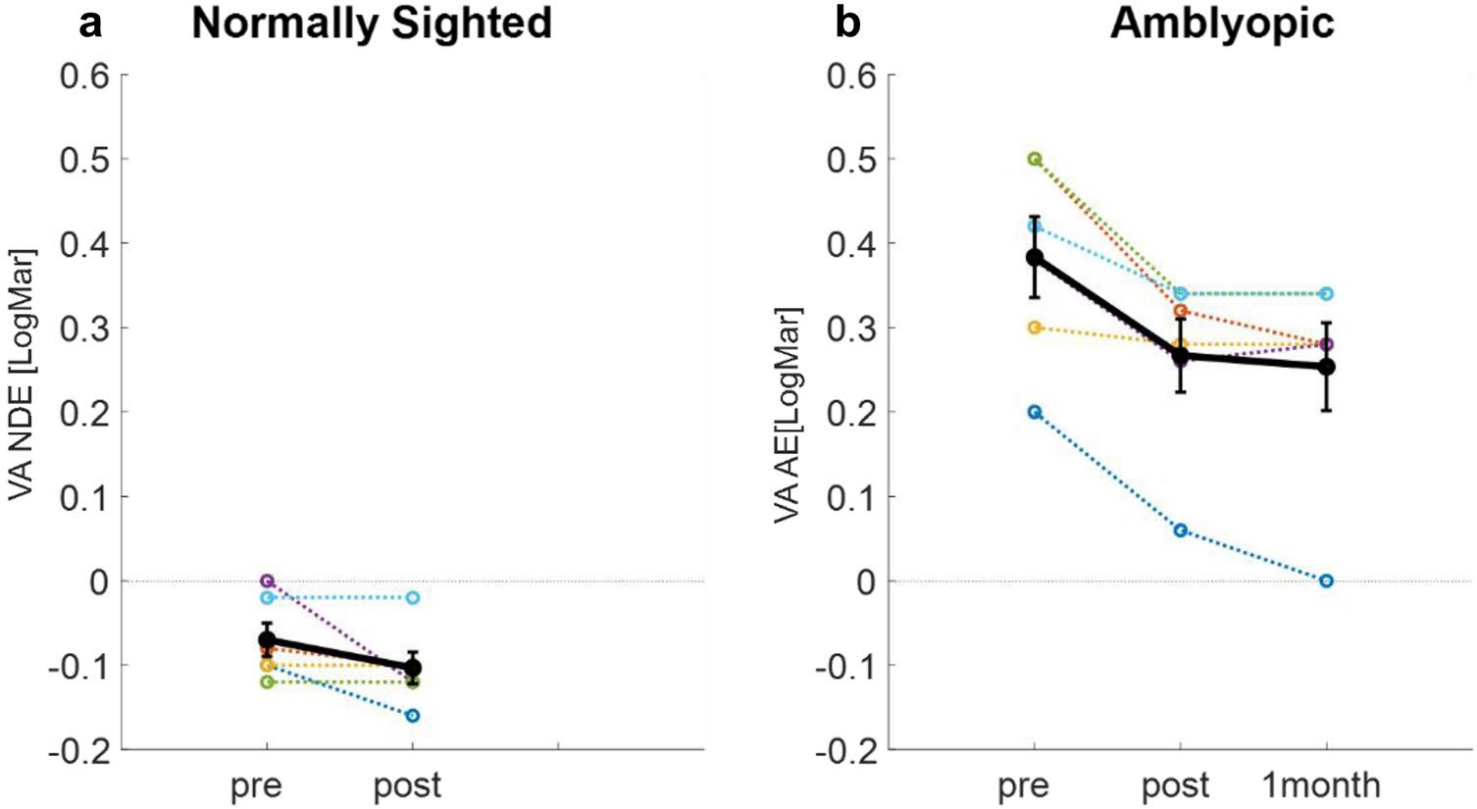
The effect of training on visual acuity (**a**) normally sighted subjects’ VA of NDE. (**b**) Amblyopic subjects’ VA of AE. VA is presented in Log Mar units. Each color denotes an individual subject. The solid black line denotes the mean VA of all 6 subjects. Error bars refer to the standard error of the mean.

Interestingly, training by a flickering stimulus induced a significant increase in the VA of amblyopic subjects by an average of 0.12 LogMar (0.38 ± 0.01 vs 0.26 ± 0.01(mean ± Std), paired two-tailed t-test *p* <  < 0.01, t(5) = 4.88) (Fig. 3). The lower than 1 slope of 0.78 (R^2^ = 0.74, *p* = 0.025) for the chart’s VA improvement suggests that the lower the initial acuity, the greater the benefit of training, similar to previous reports^76^ (Fig. S4). Importantly, the long-term effect of training on VA was evident after a month (Fig. 3).

No significant improvement was observed in the VA of the FE of amblyopic subjects, and in normally sighted subjects (0.02 ± 0.004 vs − 0.03 ± 0.00404 (mean ± SE), paired two-tailed t-test *p* = 0.2, t(5) = 1.4, − 0.07 ± 0.004 vs − 0.1 ± 0.004 (mean ± SE), paired two-tailed t-test *p* = 0.08, t(5) = 2.23 for FE and NDE, respectively). The average VA for the specific groups and eyes is presented in Table 1.

**Table 1:** VA change after training.

		Before training	Following training (average LogMar)	Change	STD	Paired T-test (pre Vs. post training)
Amblyopic	AE	0.38	0.26	0.12	0.06	P = 0.004, t(5) = 4.8
	FE	0.02	− 0.03	0.05	0.08	P = 0.2, t(5) = 1.4
	OU	− 0.01	− 0.05	0.04	0.09	P = 0.3, t(5) = 1.08
Normally sighted	NDE	− 0.07	− 0.1	0.03	0.03	P = 0.08, t(5) = 2.23
	DE	− 0.07	− 0.08	0.01	0.03	P = 0.36, t(5) = 1
	OU	− 0.1	− 0.12	0.02	0.05	P = 0.33, t(5) = 1.07

### Spatial visual function: contrast sensitivity

**Figure 4:**
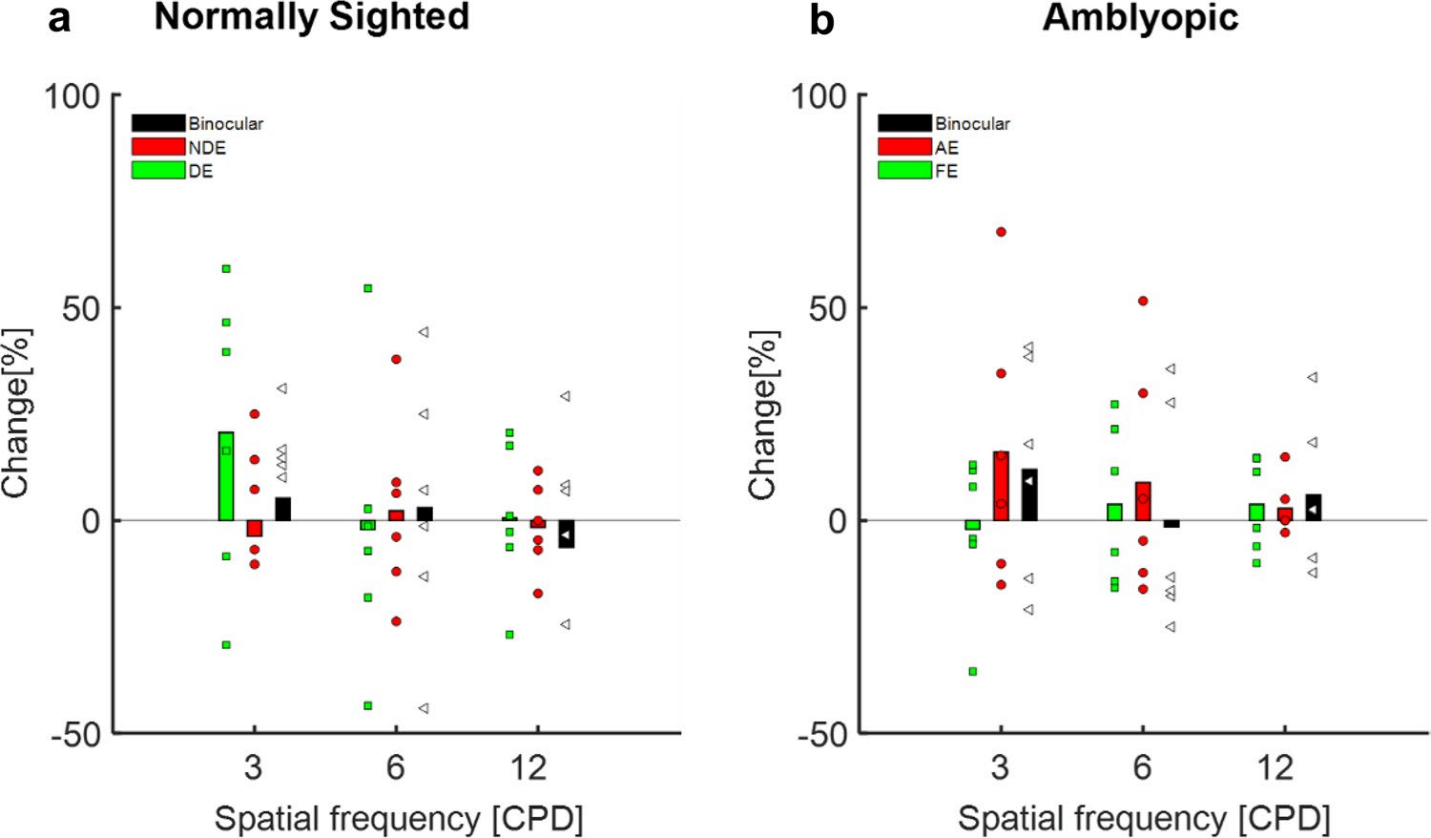
The effect of training on contrast sensitivity measured at 3 spatial frequencies: the percentage change in CS following training is depicted for various stimulus spatial frequencies (CPDs). (**a**) Normally sighted subjects. Black bars denote the mean change in CS under binocular viewing conditions, the red bars denote the mean change in CS for the NDE, and the green bars denote the mean change in CS for the DE. Each black triangle, red circle, and green square denotes the change in an individual’s CS under binocular viewing conditions, the NDE, and the DE, respectively. (**b**) Amblyopic subjects. Black bars denote the mean change in CS under binocular viewing conditions, red bars denote the mean change in CS for the AE, and green bars denote the mean change in CS for the FE. Each black triangle, red circle, and green square denotes the change in an individual’s CS under binocular viewing conditions, the AE, and the FE, respectively.

**Figure 5:**
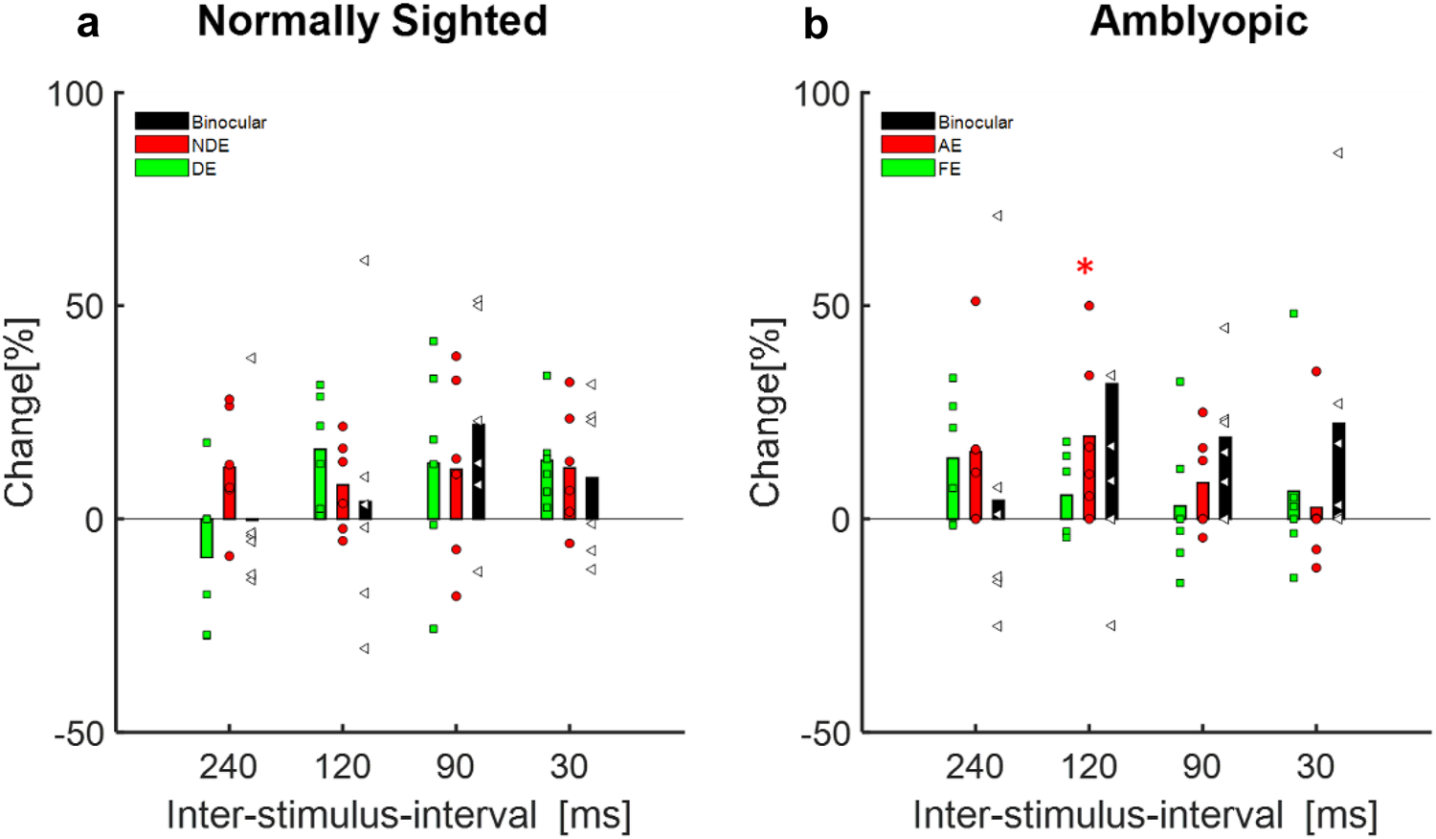
The effect of training on contrast sensitivity in a backward masking task: The percentage change in CS after training is depicted for various inter-stimulus time differences. (**a**) Normally sighted subjects. Black bars denote the mean change in CS under binocular viewing, red bars denote the mean change in CS for the NDE, and green bars denote the mean change in CS for the DE. Each black triangle, red circle, and green square denotes the change in an individual’s CS under binocular viewing conditions, the NDE, and the DE, respectively. (**b**) Amblyopic subjects. Black bars denote the mean change in CS under binocular viewing conditions, red bars denote the mean change in CS for the AE, and green bars denote the mean change in CS for the FE. Each black triangle, red circle, and green square denotes the change in an individual’s CS under binocular viewing conditions, the AE, and the FE, respectively.

In contrast to the improvement observed in VA following training, no change was found in the CS of the amblyopic and normally sighted subjects (paired two-tailed t-test *p* > 0.5) (Fig. 4). Interestingly, training induced a statistically significant improvement in the BM task, manifested by the significantly higher contrast sensitivity following training (paired two-tailed t-test *p* = 0.0027, t(23) = − 3.368 (Fig. 5).

### Training induced a significant increase in flicker stereopsis performance

Following the training-induced improvement observed in temporal (CFF) and the spatio-temporal (BM) functions of the AE, we hypothesized that the increase in the temporal function of the AE will lead to better synchronization between the two eyes, therefore enhancing binocular function.

To further evaluate the effect of training on visual function, we assessed stereo-acuity using three stereoacuity tests: the ‘Randot’ test, computerized stereopsis, and the flickering stereopsis test. The flickering stereopsis is performed by presenting flickering stereo images that are introduced to the eyes at a rate of 5 Hz. The stereo threshold (disparity angle) is evaluated using the 2AFC staircase paradigm (see the “Methods”); lower thresholds suggest a better stereopsis function. We developed this paradigm in order to evaluate the effect of inter-ocular synchronization on binocular functions, such as stereoacuity. In contrast to conventional static stereoacuity tests, stereo perception, elicited by the flickering images presented to the subjects in this paradigm, is dependent on the eyes’ temporal synchronization.

Indeed, training elicited a significant improvement in the flickering stereopsis task for the amblyopic subjects (618.4′′ ± 163′′ vs 533.6′′ ± 163′′ (mean ± SE), an average gain of 85 arcsec, paired two-tailed t-test *p* = 0.048, t(5) = 1.95).

**Figure 6:**
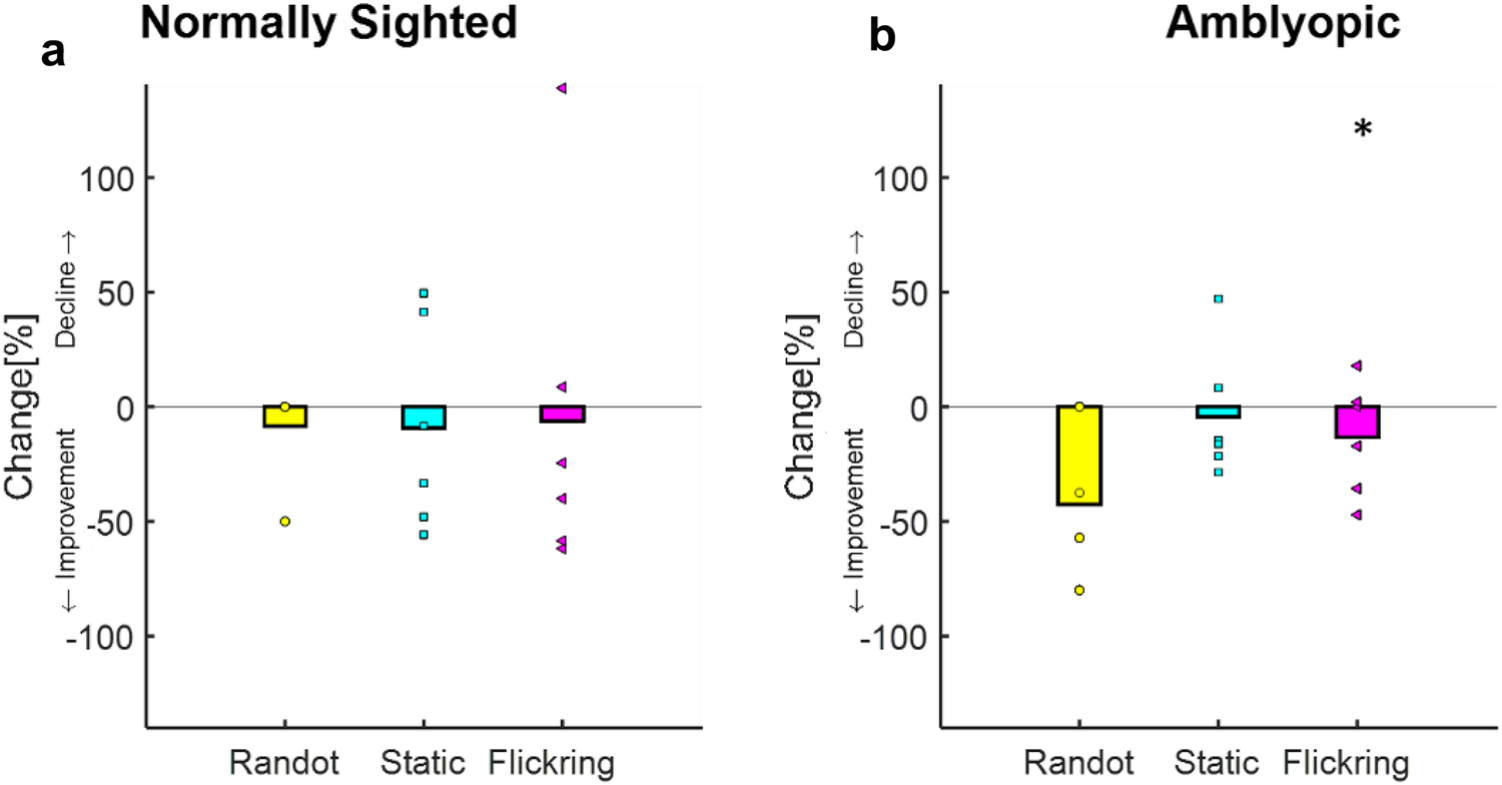
The effect of training on static and flickering stereopsis. (**a**) Stereopsis thresholds’ percentage change in normally sighted subjects following training. (**b**) Stereopsis thresholds’ percentage change in amblyopic subjects after training. The yellow bars denote the mean “RanDot” test change, the cyan bars denote the mean computerized stereopsis test change, and the magenta bars denote the mean temporal computerized stereopsis (at 5 Hz) change. Each yellow circle, cyan square, and magenta triangle denotes an individual’s change in stereopsis threshold for “RanDot”, computerized stereopsis, and temporal computerized stereopsis (at 5 Hz) change, respectively.

Although the two static stereo function tests showed an improvement trend in amblyopic subjects following training, the difference was not statistically significant (280′′ ± 89′′ vs 137′′ ± 67′′ (mean ± SE), paired two-tailed t-test *p* = 0.07, t(5) = 1.74 for the ‘Randot’ test and (409.3′′ ± 188′′ vs 348.04′′ ± 136′′ (mean ± SE), paired two-tailed t-test *p* = 0.1, t(5) = 1.44 for the static computerized task). Normally sighted subjects showed a small but non-statistically significant change after training in all tasks (3 arcsec improvement, paired two-tailed t-test *p* = 0.18 t(5) = 1, 14 arcsec, *p* = 0.14 t(5) = 1.2, 35 arcsec *p* = 0.15 t(5) = 1.2 for the ‘Randot’ test, the static computerized task, and the flickering stereopsis task, respectively) (Fig. 6).

### Training significantly increased the amplitude of ssVEP and increased the inter-ocular synchronization

Following the psychophysical investigation, we proceeded with a more objective electrophysiological evaluation by recording steady-state VEP in response to a flickering stimulus of 15 Hz under both monocular and binocular viewing conditions. We evaluated the effect of training by comparing the pre- to the post-training VEP amplitudes.

**Figure 7:**
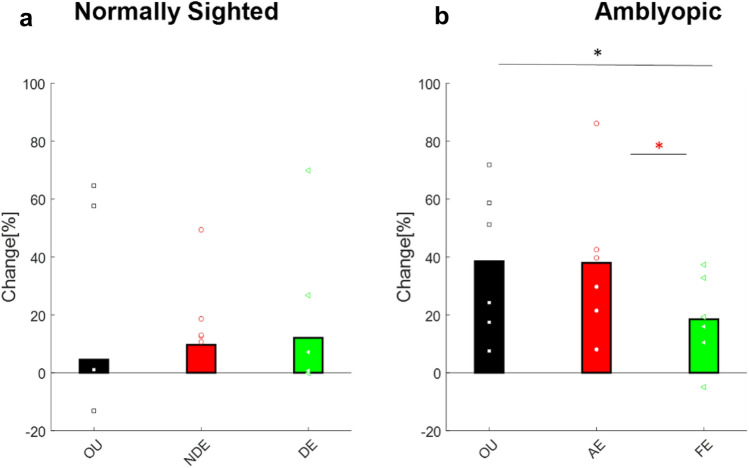
The effect of training on ssVEP: percentage change in VEP amplitude following training. (**a**) Normally sighted subjects. Black bars denote the mean change in VEP amplitude under binocular viewing conditions, red bars denote the mean change in VEP amplitude for the NDE, and green bars denote the mean change in VEP amplitude in the DE. Each black square, red circle, and green triangle denotes an individual’s change in VEP amplitude under binocular viewing conditions, the NDE, and the DE, respectively. (**b**) Amblyopic subjects. Black bars denote the mean change in VEP amplitude under binocular viewing conditions, red bars denote the mean change in VEP amplitude for the AE, and green bars denote the mean change in VEP amplitude for the FE. Each black square, red circle, and green triangle denotes an individual’s change in VEP amplitude under binocular viewing conditions, the AE, and the FE, respectively .

Training significantly increased the VEP amplitude of the AE (2.63 ± 0.18 vs 3.54 ± 0.23 paired two-tailed t-test: p = 0.008, t(5) = − 4.17, Fig. 7b). Training was also associated with a mild increase in the VEP amplitude of the FE and OU viewing conditions; however, the effect was not statistically significant (3.4 ± 0.7 vs 3.8 ± 0.8 paired two-tailed t-test p = 0.66, t(5) = − 0.4, 2.8 ± 0.6 vs 3.6 ± 0.6 paired two-tailed t-test p = 0.33, t(5) = − 1.07, respectively). Similarly, in normally sighted subjects, training was associated with a trend of an increase in VEP amplitudes; however, the effect was not statistically significant (3.17 ± 0.4 vs 3.5 ± 0.5 paired two-tailed t-test p = 0.46 t(5) = − 0.8, 3.12 ± 0.2 vs 3.45 ± 0.5 paired two-tailed t-test p = 0.52 t(5) = − 0.7, 4.01 ± 0.9 vs 4.03 ± 0.8 paired two-tailed t-test p = 0.8, t(5) = 0.27 for NDE, DE, OU, respectively) (Fig. 7a).

**Figure 8:**
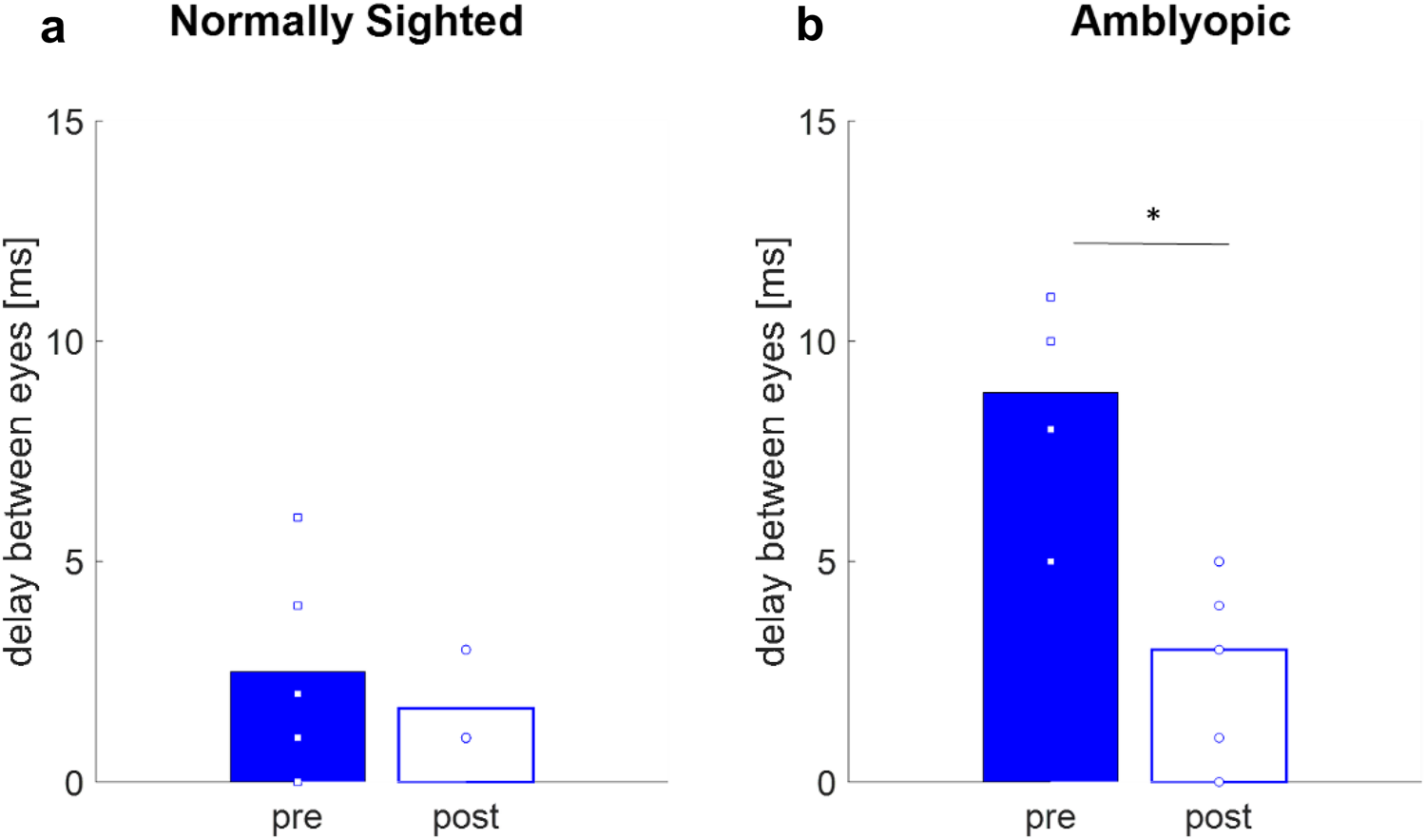
The effect of training on inter-ocular synchronization: temporal delays between eyes were estimated by the cross-correlation peak of the VEP signals arising from each eye. Blue bars denote the pre-training mean delay between eyes and white bars denote the post-training mean delay between eyes. Each blue square denotes one subject’s pre-training and each circle denotes one subject’s post-training. (**a**) Normally sighted. (**b**) Amblyopic subjects. Statistical significance is indicated: * p <  = 0.05.

As a measure of ocular functional synchronization, we calculated the inter-ocular phase using the cross-correlation method^77^. As expected, before training, the inter-ocular phase in amblyopic subjects was significantly longer compared with that of the normally sighted subjects (8.8 ± 0.9 ms vs 2.6 ± 0.8 ms, two-sample t-test *p* = 0.001, t(10) = 4.57), suggesting significantly lower inter-ocular synchronization in amblyopic subjects, compared with normally sighted subjects. Following training, the inter-ocular phase in amblyopic subjects improved dramatically to 3 ms, on average (paired two-tailed t-test *p* = *0.01 t(5)* = *3.85*), a value comparable to that of normally sighted subjects (two-sample t-test *p* = *0.2488, t(10)* = *0.13)* and was not significantly different from the values for normally sighted subjects after training (Fig. 8)*.*

**Figure 9:**
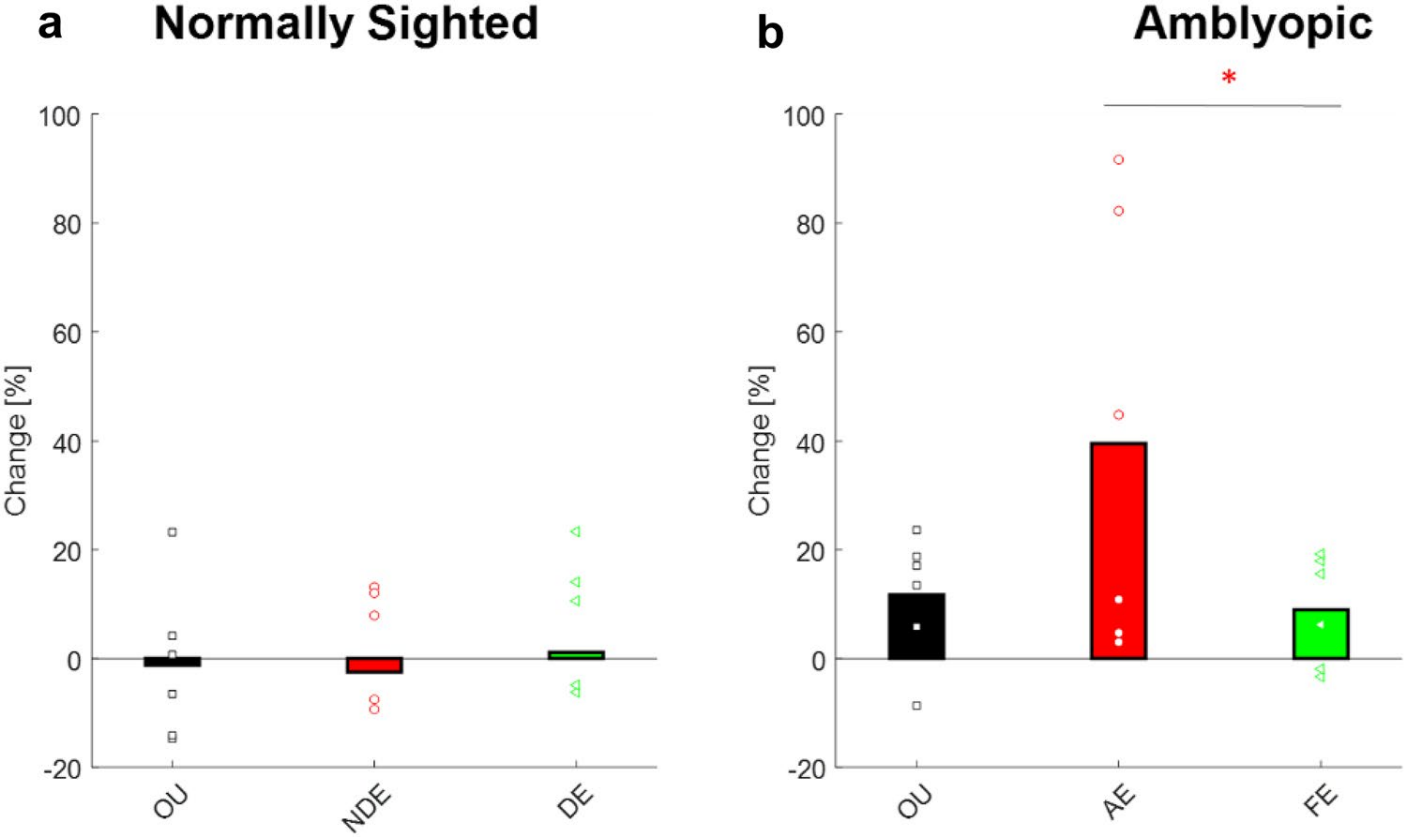
The effect of training on the signal-to-noise ratio (SNR): Percentage change in SNR following training. (**a**) Normally sighted subjects: black bars denote the change in VEP SNR under binocular viewing conditions, red bars denote the change in VEP SNR for the NDE, and green bars denote the change in VEP SNR for the DE. Each black square, red circle, and green triangle denotes an individual’s change in VEP SNR amplitude under binocular viewing conditions, the NDE, and the DE, respectively. (**b**) Amblyopic subjects: black bars denote the change in VEP SNR under binocular viewing conditions, red bars denote the change in VEP SNR in the AE, and green bars denote the change in VEP SNR in the FE. Each black square, red circle, and green triangle denotes an individual’s change in VEP SNR amplitude under binocular viewing conditions, the AE, and the FE, respectively. Statistical significance is indicated: * p <  = 0.05.

As an additional measure for studying the effect of training on the recorded VEP, we evaluated the signal-to-noise ratio (SNR). Before training, the SNR of the AE was significantly lower, compared with that of the FE (14.5 ± 0.05 vs 19.6 ± 1.9, two-sample t-test *p* = *0.006, t(10)* = − *2.76*) and NDE (two-sample t-test *p* = *0.003 t(10) − 3.829*) (Fig. 9), suggesting significantly higher noise in the amblyopic eye, compared with the FE and with normally sighted subjects (Fig. S5).

Following training, the SNR in amblyopic subjects improved (increased) dramatically by an average of 39% (14.5 ± 0.05 vs 18.9 ± 1.4 paired two-tailed t-test *p* = *0.024, t(5)* = *− 2.58*), to levels that are comparable to those of the normally sighted subjects (18.9 ± 1.4 vs 17.5 ± 0.98 two-sample t-test *p* = *0.32, t(10)* = *0.76).* In contrast, no effect of training on the VEP SNR of normally sighted subjects was observe (Fig. 9) (17.9 ± 0.35 vs 17.5 ± 0.98, 19.5 ± 0.42 vs 19.44 ± 1.09, 24.87 ± 0.9 vs 24.4 ± 1.02, DE, NDE, OU, respectively).

## Discussion

Over recent decades, there has been a growing interest in the use of vision training to treat amblyopia^59^; the focus of this type of training is primarily on improving spatial visual performance while using mainly stimuli with spatial features. The current research focused on improving the temporal function by using pure temporal (flickering) stimuli; we also evaluated whether the temporal improvement will be transferred to the spatial domain and whether the interocular integration will be enhanced.

During 5 training sessions, the amblyopic eye’s CFF threshold consistently and significantly improved by 2.4–4.7 Hz (up to 17.2%), eventually reaching levels that were not significantly different from those of the normal fellow eye. The magnitude of improvement reported in studies^43,78^, where normal subjects were trained by motion targets is comparable to the improvement observed in our amblyopic subjects (2–6 Hz); however, in these studies the training procedure was significantly longer (1 h, 9 sessions), compared with our procedure (± 30 min, 5 sessions). It could be possible that the longer training session or more complex stimuli would enhance CFF in the normally sighted subjects.

The strong evidence of the success of vision training lies in the generalization of the improvement to other visual tasks^34^; the more transfer found in amblyopes is indicative of greater visual plasticity in amblyopic subjects, compared with normally sighted subjects^36,79,80^. In previous review on PL^36^ it is stated that greater generalization is obtained through shorter training sessions, whereas extensive training with a fixed stimulus may lead to over-specificity of training. Indeed, here we employed a short training procedure aimed at improving temporal visual functions and found a generalization of training, as was evident with a significant improvement in VA, BM, and stereopsis.

The average VA magnitude of improvement (0.12 LogMar) observed in the amblyopic group is comparable to previous studies^32^. Importantly, the magnitude of improvement was dependent on the baseline VA (see Fig. S4), consistent with findings in previous studies^32,81^. It should be mentioned that in the current research the amblyopic group’s visual acuity impairment was relatively moderate (VA = 0.2–0.5 LogMar, 0.38 LogMar on average—mild amblyopia); thus, the expected improvement is moderate. The ratio of post (training) VA to pre (training) VA (the so-called PPR, post:pre ratio) is often used to quantify the correlation of VA improvement with the baseline VA. In our study the average of this ratio was found to be 0.67, comparable to the ratios reported in previous studies^82^. Importantly, the long-term effect of training on VA was evident after one month. However, future studies should evaluate the long-term effect over a longer period of time, and whether there is a need for periodic re-training.

Improvement in the temporal performance of the AE was also observed for the Backward Masking task, which is considered a spatio-temporal visual function. The larger improvement observed in the AE was 19.4%, found for 120 ms. In a previous work we showed that training with a contrast Gabor target and a BM paradigm increased both CS, BM, and was also associated with an increase in the processing speed^57,83^; the increase in the processing speed was further generalized to an improvement in untrained functions, such as crowding and detection. It could be possible that in the current study the improvement in the processing speed is the mediating factor for generalization.

In contrast with the improvement in the above-mentioned visual functions, there was larger variability of the effect of training on the non-trained tasks of CS and CFF measured at various luminance; these effects were not statistically significant and could be caused by natural variability in non-trained subjects. Future studies will further explore the effect of training on these visual functions.

Previous studies questioned whether or not the improvement is mainly due to eye occlusion during training^84^. It is highly unlikely that this is the underlying mechanism for the observed improvement in our PL paradigm; first, because it is a relatively short training procedure (5 sessions) and patching for such a short time (on average, a total of 150 min over 2.5 weeks) does not usually affect the visual acuity^84^. Second, the experiment was performed using dichoptic glasses such that although the FE was not trained, it was not patched and was open during the training^84^.

The improvement in CFF and VA was further accompanied by an improvement in stereopsis in amblyopic subjects. Stereo acuity is an important measure of the ability of both eyes to work and synchronize with each other. Moreover, improving stereo-acuity is an important measure of the success of PL generalization. Recent work^67,85^ suggests that amblyopic subjects may regain binocular function if the signals from the two eyes are appropriately balanced. We believe that PL facilitated the gain in the amblyopic eye, as manifested by the increase in both CFF and spatial vision; this is supported electrophysiologically by an increase in VEP amplitude observed in our amblyopic subjects. Electrophysiological studies in animals and humans are in agreement with our results, showing that training induced modification of activity in local connections within V1^86–91^. It is worth noting that these studies have dealt with spatial learning and here we present our novel results in a “pure” temporal task, showing a similar outcome of training. Another objective measurement observed in amblyopes is reduced SNR, possibly related to desynchronization of neuronal activity. Our results indicated that the amblyopic eye had significantly lower SNR, compared with the fellow eye and that this measure improved dramatically after training; these results are in agreement with studies in the field of amblyopia treatment (e.g.^92^).

PL further enhanced the interocular synchronization, as manifested by a significant reduction in the inter-ocular phase delay, 8.8 ms ± 2.3 Vs. 3 ms ± 2.8 following training. A change in interocular synchronization, following treatment of the amblyopic eye, is in agreement with previous studies^92^. Indeed, balancing and synchronization information arriving from the two eyes was followed by a significant improvement in flickering stereopsis functions.

Although we show here novel and important results, our study has several drawbacks. First, our amblyopic subjects were of diverse amblyopic aetiology, consequently increasing the training effect’s variability; a different amblyopic aetiology may affect the training effect^82^. Nevertheless, it should be mentioned that CFF, VA, BM, and stereopsis improved following training for both the anisometropic and strabismic subjects. Future studies should increase the sample size and enable comparisons of more homogeneous groups and inter-groups. Furthermore, the training procedure consisted of only one specific training procedure; thus, it did not enable comparing various procedures or studying a dose effect of training duration, which could enable optimizing learning.

In conclusion, we report here a significant improvement in temporal and spatial vision in amblyopes following a simple short training procedure based on flickering stimuli. VEP recording also showed that training increased the visual cortex amplitude responses to flickering light and increased the interocular synchronization. The increase in interocular synchronization was further manifested by a significant increase in flickering stereopsis. These findings advance our understanding of the complex processes underlying perceptual learning of both temporal and spatial vision as well as learning transfer. Although further work is still needed to optimize this method for clinical applications, these results highlight the potential of a simple flickering stimulus PL to improve both temporal and spatial visual performance. A future mobile version of the device can increase trianing availability by enabling at-home training. The results also shed light on the process of inter-ocular balance and synchronization, which is critical for functional binocular vision.

## Methods

### Subjects

PL and measurements were performed on a total of 12 subjects: 6 normally sighted subjects (4 females, 2 males aged 22.1 ± 1.5 years old, mean ± STD) with no known neurological conditions and with normal corrected vision, and 6 amblyopic subjects (5 females, 1 male, aged 28.9 ± 7.1 years old, mean ± STD). Group size was estimated based on our previous subjects’ data distribution using a standard calculation method^93^.

All subjects underwent a comprehensive eye examination by a qualified optometrist (A.E.E.), similar to previous works of our group^26,73^. Subjects were refracted by dry retinoscopy and tested using a binocular ‘Randot stereo’ test, a cover-test, and underwent a general eye examination including fundus ophthalmoscopy and a slit lamp examination of the anterior segment. We evaluated best-corrected visual acuity (BCVA) using the gold-standard ETDRS chart^94^. The criteria for normally sighted subject inclusion were visual acuity (ETDRS chart) better than 0.1 LogMar with a difference of less than 0.2 LogMar between eyes, stereopsis better than 40″, and no ocular or neurological diseases. The mean stereopsis of the subject groups was better than 40″, and the mean visual acuity (LogMar) was as follows: far monocular: − 0.07, far binocular: − 0.1. For amblyopic subjects, the criteria for inclusion were a difference between the eyes of at least 0.2 LogMar and an indication of the presence of strabismus or a difference in the refractive error. (The details of the visual parameters of the amblyopic subjects appear in Table S1 in the Supplementary Material).

### Training procedure

All subjects were fully corrected, and a three-month refractive adaptation period was applied if necessary.

Subjects underwent 5 monocular training sessions (on average, a total of 150 min over 2.5 weeks) of the amblyopic eye in amblyopic subjects or the non-dominant eye in the normally sighted subjects; each session included a task similar to the CFF test using the method of constant stimuli in a 2AFC paradigm. The frequency range of the random flickering stimulus during training was determined according to CFF thresholds, which were recorded as part of the baseline evaluation for each subject. Since studies have shown that training around the threshold is more effective^95,96^, the range for training was determined to be between 5 Hz below and 5 Hz above the CFF threshold. In a training session each frequency condition was repeated 20 times^97^; an auditory tone was presented when a mistake was made. Subjects were allowed to rest as needed during the training sessions. The psychometric curve was then plotted as a function of the flickering frequency against the percent correct. The threshold was determined after fitting the results into a sigmoid curve and calculating the frequency yielding 80% correct.

### Visual function tests

Comprehensive visual function tests were performed before and after training, including monocular and binocular CFF, visual acuity, contrast sensitivity, backward masking, and stereopsis (‘RanDot’, static, flickering stereopsis).

#### CFF measurements

In addition to the CFF measured during training sessions in a monocular condition using the constant stimuli method, CFF was also measured monocularly and binocularly using a staircase method, similarly to our previous reports^26,73^. Briefly, the subject is seated behind linear polarizing glasses mounted on a chin rest. Both the glasses and the LED filter are orthogonally polarized so that each eye is stimulated by only one LED. In order to create binocular testing conditions, with characteristics similar to the monocular examination, we designed the system apparatus such that the two LEDs were perceived as a single LED.

The experiment begins with a dark adaption period of 3 min (a mean room luminance of 0.001 cd/m^2^), which was found to be enough for CFF testing using our approach^73^. During the adaptation time, the experimental procedure was explained to the subject. Experiments were designed to evaluate the CFF threshold through a psychophysical test, based on a discrimination task using the two-temporal alternative forced-choice paradigm (2TAFC) with a stimulus duration of 1 s, similar to our previous studies^26,73^. The target stimulus temporal features were modulated using the staircase method, as described below. Subjects were requested to discriminate between a target stimulus (a flickering light at various frequencies) and a flickering light at a frequency of 120 Hz, which is significantly higher than the CFF in humans and is therefore perceived as continuous light. The staircase method was implemented by modifying the stimulus frequency using an adaptive 3:1 method according to the participant’s response^73,98^ (with fixed-step sizes of 2 Hz) of the 3-up-1-down staircase (3:1)^99^. The test was finalized upon the completion of 8 reversals (a change in the direction of the stimulus frequency); the CFF was defined as the mean of the last six reversal values, and the threshold was calculated as 79% correct^34,99^. The entire procedure was repeated twice to increase reliability and the CFF threshold was calculated as the mean of the two repetitions. The test can be performed in a combined manner such that during a specific trial both eyes are tested, and the stimulus is randomly presented to either eye or to both eyes simultaneously. The test duration is 20 min, and the results are saved for further analysis. The interface used to control the experimental paradigm and the psychophysical tests was developed using MATLAB (see previous studies for more details^26,73^).

#### Contrast sensitivity

Advanced computerized tests to examine the spatial visual perception were performed based on a procedure previously described by our group^48,100–104^. Briefly, the tests are based on a 'PSY—psychophysical tool' software including contrast sensitivity and backward masking (BM)^105^. Gabor patches at various spatial frequencies as well as contrast ISI (which is detailed for each test) were used as the visual stimuli^106^. Stimuli were presented on an 'EIZO FORIS- FG2421 “Turbo 240”' computer screen at a 100 Hz refresh rate. The high-performance 'EIZO' screen was calibrated as follows: Brightness = 100, Contrast = 50, Resolution = 1024 × 768. The screen was calibrated, and gamma corrected. The task was either to detect a Gabor with different contrast levels or to detect a Gabor target in the masking experiments.

#### Stereopsis tests

##### ‘RanDot’

In order to evaluate stereo-acuity, the ‘RanDot’™ stereo test (Stereo Optical Co., Chicago, IL) was performed at a 40 cm testing distance under standard room illumination. Polarized targets and polarizing viewers provide separate images of the targets to the two eyes. The ‘RanDot’™ stereo test is based on the random dot principle. First, a suppression check was performed, whereby the right eye sees the R and a vertical line, and the left eye sees the L and a horizontal line. The absence of one of the components indicates suppression of one eye under binocular conditions; therefore, stereo-acuity was recorded as ‘Absent’. In the second stage, the Randot Form test was performed; simple geometric forms and the letter E (500 arcsec and 250 arcsec) are central in each of 4 areas except one, which acts as a control. Subjects were asked which area does not appear to have any form in it. In the last stage, the Randot circles test was performed, testing the ability to distinguish differences at a perceived distance of static circles based on the relative disparities of the targets. Subjects were presented with contoured circles at 10 discrete disparity levels (from 20 to 400 arcsec) and were asked to choose which of the 3 circles at each disparity level appears closer than the other two—a simple 3-alternative forced-choice test. Stereo-acuity was determined as the level of stereopsis in the last row of circles chosen correctly.

##### Flicker and static stereopsis tests

The stimuli were presented on a Dell Alienware 25 Monitor AW2521HFL equipped with a GeForce GTX 1650 Graphics Card, NVIDIA. Visual stimuli were generated under the control of MATLAB with the Psychophysics Tool-box^107^ in 64-bit Microsoft Windows. The experiment was conducted in a dark room, such that the screen provided the only measurable light input to the eyes. The screen was set to a spatial resolution of 1920 × 1080 pixels with a refresh rate of 240 Hz, calibrated, and gamma corrected. At a 100 cm viewing distance, the screen provided ~ 64 pixels per degree of visual angle, and pixels subtended ~ 55′′ of arc (arcsec). The stimulus consisted of two squares (3 × 3 degrees) and was presented on a black screen with a white random dot background. Each square was composed of a combination of random red and blue dots and was static or flickered at a frequency of 5 Hz. The subject wore glasses with red and blue filters such that the red dots were presented to the right eye and the blue dots were presented to the left eye. Stereopsis depth was controlled by manipulating the disparity (between the red and the blue dots) and the subject’s task was to choose which square (left or right) protrudes in a 2AFC paradigm. The stereopsis depth was modified in a staircase (3 up 1 down) paradigm in order to find the stereopsis threshold.

#### Steady state visual evoked potentials (ssVEP)

##### VEP recordings

VEP recordings were performed similarly to our previous report^27^ using the CURRY 8X (Compumedics Neuroscan) system. Signals were amplified × 4000 (SynAmps RT 64-channel Amplifier), the sampling rate was 1.0 kHz. Electrode impedances were kept below 10kΩ. Using a custom-written MATLAB program, we processed the information from the Oz (inion) central electrodes in the occipital lobe (the reference electrode was taken as Fz). The stimulus consisted of a 15 Hz flickering light^27^ presented for 5 s using our customized device^26^. The stimulus was randomly presented to each eye separately or to both eyes. The stimulus size was 0.47 degrees.

The average ssVEP amplitude was obtained by segmenting the EEG signal using the stimulus triggers that indicated the start of the flickering. The trend in the segmented data was removed and the data were filtered with a band-pass filter with cut-off frequencies of 0.1–100 Hz. The first second was deleted from the analysis because it is highly affected by the abrupt change from dark to light at the beginning of each trial. The remaining 4 s were averaged for a wave of 15 Hz.

##### VEP cross-correlation analysis

In the current study we used a unique method for a quantitative evaluation of the inter-ocular synchronization using cross-correlation analysis. Cross-correlation is a measurement of similarity between multiple time series and in particular, it is used to find where they best match^106^. Here we calculated the cross-correlation between the right and left eye waves using the cross-correlation function of Matlab. We then estimated the interocular delay by finding the time difference between the two waves that give the highest correlation.

### Statistical analysis

A paired two-tailed t-test, as planned comparisons, was used to compare two conditions within the same subjects; the significance is reported as t and p values. A two-sample Welch t-test with a two-tailed t-test was used to compare the normally sighted and amblyopic subjects; the significance is reported as t and p values. When the p value was smaller than three zero digits, we reported it as p << 0.001.

Two-way ANOVA was used for assessing the main effects and the interactions. For comparisons with degrees of freedom higher than 1, the post hoc tests of a multiple comparison test were applied (for any ANOVA).

Analyses were performed using Matlab software (Mathworks, Waltham, MA) Statistics and Machine Learning Toolbox™, with significance set at p < 0.05. A Quantile–Quantile plot analysis showed a normal distribution of the data. All data are reported as the mean ± standard error of the mean (SE).

### Ethics approval and consent to participate

All subjects provided written informed consent. The study was approved and conducted according to the IRB Committee by the Bar-Ilan University Ethics Committee (ISU202012003), and was conducted with adherent to the tenets of the Declaration of Helsinki.

## Supplementary Information

Supplementary Information.

## Data Availability

The datasets during and/or analyzed during the current study available from the corresponding author on reasonable request.
