# Supplementary material for: Perceptual learning based on a temporal stimulus enhances visual function in adult amblyopic subjects
Source: Sci Rep. 2023 May 11;13:7643. doi: 10.1038/s41598-023-34421-3 (PMC10175483; doi:10.1038/s41598-023-34421-3)
Supplement: Supplementary file 1 — Supplementary Information. [file 41598_2023_34421_MOESM1_ESM.docx]

## Supplementary Information

***Supplementary Material to the Results***

Figure S 1**: Individual psychophysical curves of the first and fifth session of training of 6 amblyopic subjects**. A right shift of the curve on the fifth day compared to the first day can be seen in all subjects’ curves, indicating an improvement in threshold.

Figure S 2: **Individual psychophysical curves of the first and fifth session of training of 6 normally sighted subjects**.


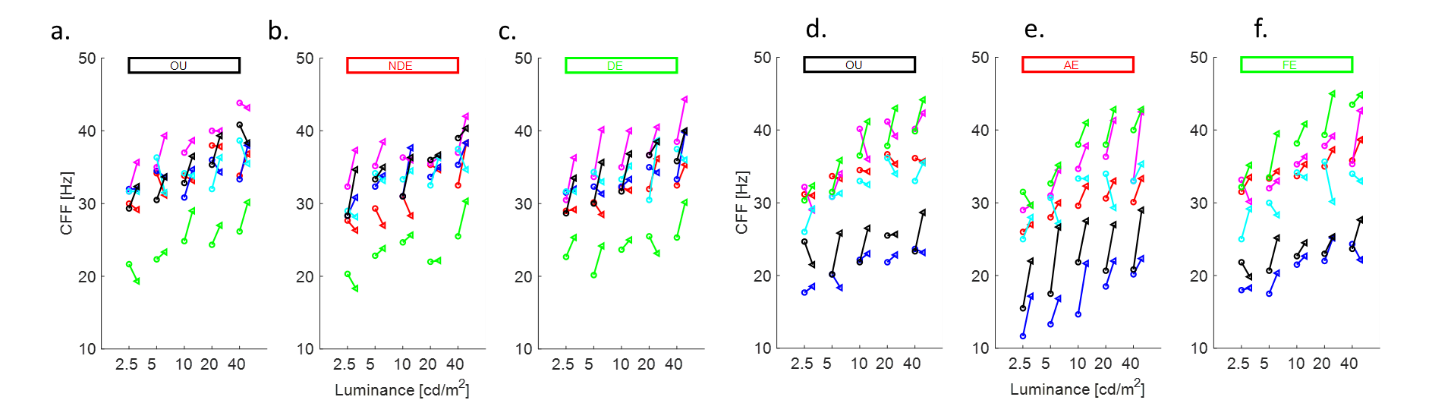


Figure S 3 **Effect of training on CFF**: **Individual changes**, Each color represents a different subject, circle represents pre training CFF, triangle represents post training CFF. (a) CFF for normally sighted under binocular viewing conditions (b) CFF of the NDE for normally sighted (c) CFF of the DE for normally sighted (d) Amblyopic subjects CFF under binocular viewing conditions (e)CFF of the AE for Amblyopic subjects (f) CFF of the FE for Amblyopic subjects

Figure S 4 :**Correlations between visual acuity of AE change post training and VA pre training.** Each dot represents one subject. y=0.78x-0.03, r=0.74, p=0.025


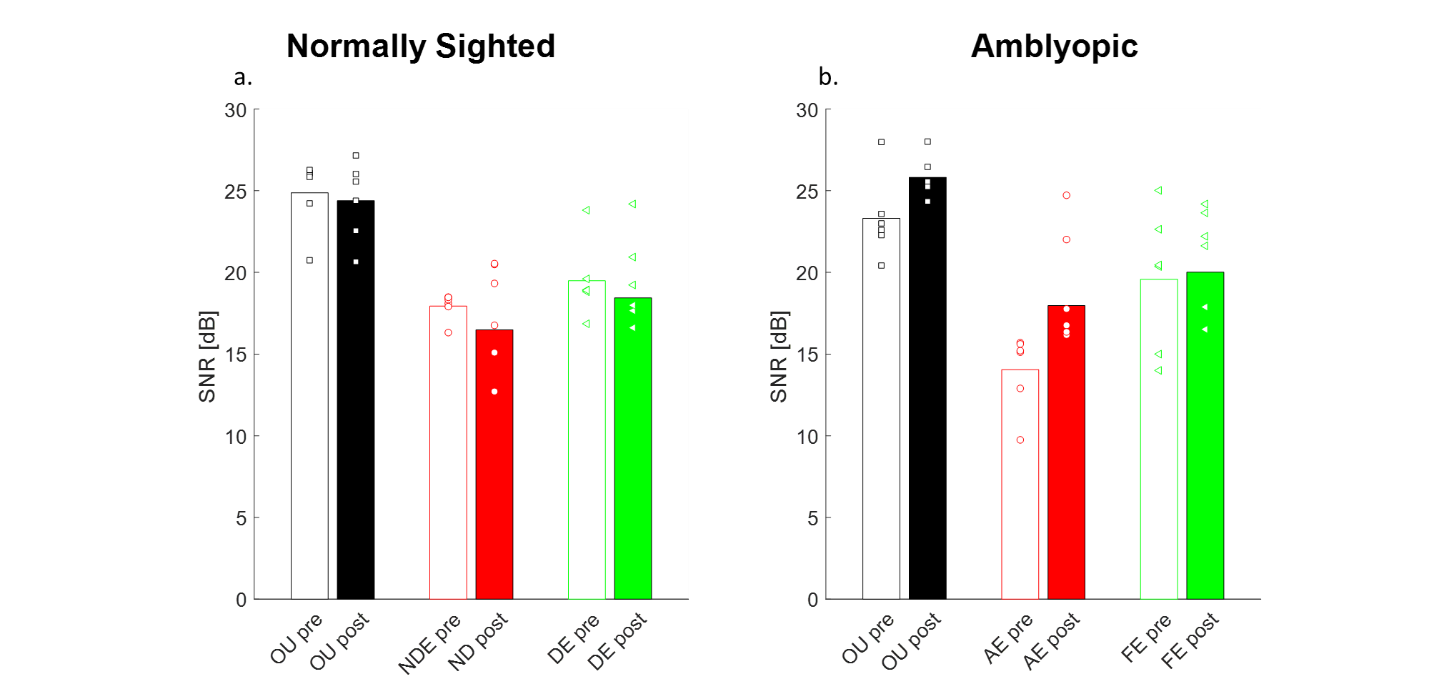


Figure S 5 **Effect of training on Signal-to-noise ratio (SNR)** (a) normally sighted subjects: Black bars represent SNR under binocular viewing conditions, red bars represent mean SNR for the NDE and green bars represent mean SNR for the . Each black square, red circle and green triangle represents one subject’s mean SNR under binocular viewing conditions, the NDE and the DE respectively (b) Amblyopic subjects. Black bars represent mean SNR under binocular viewing conditions, red bars represent mean SNR for the AE and green bars represent mean SNR for the FE. Each black square, red circle and green triangle represents one subject’s change in mean SNR under binocular viewing conditions, the AE and the FE respectively. Statistical significance was indicated:* p<=0.05

***Supplementary Material to the Methods***

Details of the visual parameters of the amblyopic subjects


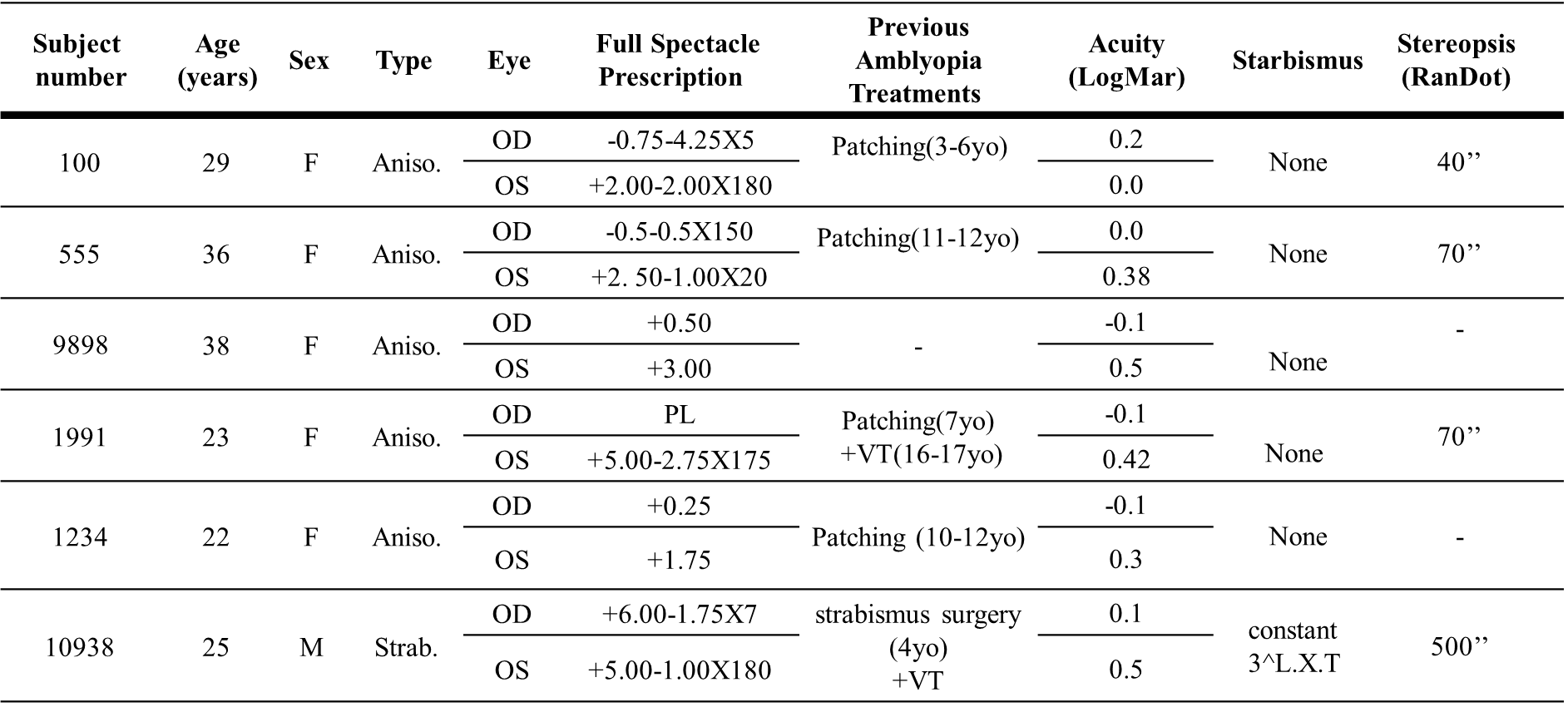


Table S 1 Details of the visual parameters of the amblyopic subjects
